# Guillain–Barre Syndrome following SARS-COVID-19 Infection: A Case Report from India

**DOI:** 10.1155/2021/4676659

**Published:** 2021-05-27

**Authors:** Abhishek Singhai, Akshit Budhiraja

**Affiliations:** Department of Medicine, All India Institute of Medical Sciences, Bhopal, India

## Abstract

Severe acute respiratory syndrome coronavirus 2 (SARS-COVID-19) is a novel coronavirus that started in Wuhan City in China in December 2019. It can cause acute respiratory infection. Guillain–Barre syndrome (GBS) is an autoimmune disease characterized by rapidly progressing ascending paralysis that is triggered by an infection or immune stimulation which produces an abnormal immune response that targets peripheral nerves. In most cases, it is preceded by a bacterial or viral infection. This is a case of a 36-year-old male patient from India who developed progressive acute flaccid paralysis after SARS-COVID-19 infection. Clinical examination and lab studies lead to the diagnosis of GBS. The patient was treated with intravenous immunoglobulins and supportive treatment. Following treatment, there was a substantial improvement in weakness as reported by the patient and was confirmed by clinical evaluation. This is an uncommon manifestation of SARS-COVID-19 infection and suggests the presence of an immune-mediated process leading to damage of the nervous system.

## 1. Introduction

Severe acute respiratory syndrome coronavirus 2 (SARS-COVID-19) virus is a positive single-strand RNA virus belonging to the family Coronaviridae and the genus Betacoronavirus, closely related to the severe acute respiratory syndrome (SARS) virus and the Middle East respiratory syndrome (MERS) virus. Disease caused by SARS-COVID-19 is named COVID-19. SARS-COVID-19 originated and spread from Wuhan city, Hubei Province in China, in December 2019 to worldwide. Globally, as of 11 May 2021, there have been 158,651,638 confirmed cases of COVID-19, including 3,299,764 deaths, reported to the WHO. In India as on 11 May 2021, there have been 2,02,82,833 confirmed cases of COVID-19, including 2,22,408 deaths with 1.10 case fatality rate [1]. SARS-COVID-19 has a characteristic spike protein on its envelope which helps in binding to the cellular receptors. It binds with the angiotensin-converting enzyme 2 (ACE2) receptors on human cells to facilitate entry into the cells. Most commonly, it causes flu-like symptoms such as dry cough, sore throat, fever, and difficulty in breathing in patients. It may also cause other symptoms such as muscular pain and gastrointestinal symptoms such as diarrhoea, nausea, and vomiting. In severe cases, it may progress to acute respiratory distress syndrome requiring initiation of mechanical ventilation and intensive care unit (ICU) care. In few studies, SARS-COVID-19 is found to cause neurological symptoms including acute cerebrovascular diseases and impaired consciousness [2]. A case of acute necrotizing encephalopathy as a complication of COVID-19 has been reported from India [3]. There have been a few reports of Guillain–Barre Syndrome (GBS) in patients of SARS-COVID-19 infection from other parts of the world [4–6]. GBS is a neurological disease characterised by acute to subacute onset of bilateral and relatively symmetrical ascending flaccid limb weakness with decreased or absent deep tendon reflexes [6]. GBS is caused by an aberrant immune response targeting various sites on peripheral nerves. The abnormal immune response is triggered by a preceding viral or bacterial infection. Molecular mimicry between the nerve antigens and the microbial agent is responsible for the abnormal immune response. The predominant infection reported in patients of GBS is *Campylobacter jejuni*. Other infections associated with GBS include Cytomegalovirus, Epstein–Barr virus, Influenza A virus, etc. The disease is characterised by the onset of weakness 3 days to 6 weeks after the preceding infection with or without sensory involvement. Weakness generally begins in lower limbs and follows an ascending pattern. The weakness progresses over a period of 12 hours to 48 days. It is associated with diminished or absent deep tendon reflexes. It may be associated with sensory symptoms like pain and paraesthesia and autonomic dysfunction. Management of GBS includes supportive treatment, including ICU care and mechanical ventilation. Definitive management includes treatment with intravenous immunoglobulin given in a dose of 0.4 g/kg/day for 5 days and plasma exchange. The definite management should be started as soon as possible to prevent irreversible nerve damage. Here, we are presenting a case of GBS in a patient following SARS-COVID-19 infection.

## 2. Case History

A 36-year-old male patient was admitted with a complaint of weakness of bilateral lower limbs associated with pain and tingling sensation for 3-4 days. Two weeks ago, he also had fever, malaise, and sore throat lasting 3 days. His nasopharyngeal swab reverse transcription-polymerase chain reaction (RT-PCR) was positive for SARS-COVID-19 infection. Weakness of limbs started 7 days after recovery from upper airway symptoms. Over the next 3-4 days, the severity of lower limb weakness increased along with the involvement of the upper limb. On examination, the patient was conscious and oriented to time, place, and person. Higher mental functions were normal. In motor system examination, the patient had a power of 1/5 in the bilateral hip and knee joints in all muscle groups and a power of 3/5 in the bilateral shoulder, elbow joints, and small muscles of hands. Deep tendon reflexes were absent in bilateral knee and ankle and in biceps, triceps, and supinator. Plantar reflex was absent bilaterally. There was no sensory deficit. No bowel and bladder involvement were observed. There was no evidence of cranial nerve involvement. Other investigation reports were as follows: hemoglobin 12.6 g/dl, total leukocyte count 10870/*µ*L, platelet count 642000/*µ*L, liver function tests within normal limits, renal function tests within normal limits, serum C reactive protein 207.47 mg/dL, serum calcium 9.18 mg/dL, serum ferritin 178 ng/ml, HbA1C 6.4%, and MRI brain and spine normal. Cerebrospinal fluid analysis showed glucose level 78.94 mg/dL, proteins 33 mg/dL, and <5 cells/cumm on smear examination. Antiganglioside antibody test could not be performed due to nonavailability at our centre.

Nerve conduction study was suggestive of demyelinating polyradiculoneuropathy with prolonged distal motor latencies, decreased amplitudes of compound muscle action potential, slow nerve conduction velocities, and increased F-wave latencies (Table 1).

The patient was diagnosed with GBS (postinfectious). The patient was treated with intravenous immunoglobulin (IVIg) in a dose of 0.4 g/kg/day for 5 days. Other treatments given included injection amoxicillin-clavulanate 1.2 g 8 hourly, injection dexamethasone 8 mg daily, and supportive treatment. After 5 days of IVIg treatment, the patient reported improvement in weakness in both upper and lower limbs. On examination, the patient had a power of 4/5 in the bilateral knee and hip joints and a power of 5/5 in the bilateral shoulder, elbow joints, and small joints of bilateral hands. The last follow-up was one month after discharge, and at that time, the patient had complete motor power recovery without residual sensory or autonomic dysfunction.

## 3. Discussion

This is a case of GBS caused by an abnormal immune reaction after SARS-COVID-19 infection. GBS was diagnosed on the basis of typical clinical features, albumin-cytological dissociation in CSF, and demyelinating polyradiculoneuropathy on nerve conduction study. GBS occurs due to molecular mimicry between the nerve antigens and inciting agents. The disease had a monophasic course with maximum weakness after almost a week of onset of the disease. Similar other cases of GBS following SARS-COVID-19 infection have also been reported. A study by Rahimi reported a case of acute motor/motor-sensory axonal neuropathy (AMSAN) variant of GBS following COVID-19 infection [5]. Another study in Italy reported 4 cases of GBS which were positive for COVID-19 at the onset of neurological symptoms [6]. Similar patients with neurological symptoms were also reported following infection with the closely related MERS-CoV and SARS-CoV [7, 8]. It has been established that various mechanisms are responsible for the neurological manifestations of SARS-COVID-19 infection. SARS-COVID-19 can directly enter into the neurons and glial cells by binding to the ACE2 receptors and cause damage [9]. SARS-COVID-19 can also cause an immune response which causes tissue damage. The exact mechanism of GBS in SARS-COVID-19 infection is unclear, and further research should be conducted for a better understanding of the disease. In this case, the patient was treated with intravenous immunoglobulin, and the patient reported a substantial improvement in symptoms following treatment. Abu-Rumeileh et al. reviewed 73 cases of COVID-19-associated GBS and found the clinical picture resembling that of classic GBS or Zika-associated GBS. They concluded that the chronological evolution, the response to IVIG, and the absence of SARS-COVID-19 RNA in CSF may suggest a prominent postinfectious immune-mediated mechanism rather than a parainfectious one [10]. Carrillo-Larco et al. reviewed eight case reports of GBS and COVID-19 and found that symptoms started between 5 and 24 days after those of COVID-19. The protein levels in cerebrospinal fluid samples ranged between 40 and 193 mg/dl. None of the cerebrospinal fluid samples tested positive for COVID-19. Six patients debuted with ascendant weakness and three with facial weakness. Five patients had favourable evolution, four remained with relevant symptoms or required critical care, and one died; the Miller Fisher case had successful resolution [11]. Kamel et al. also reported a case of GBS following COVID-19 in Kuwait having a favourable outcome with IVIG with no autonomic or respiratory affection [12]. Sheikh et al. had done a systematic review of 94 cases, and they showed that the clinical picture of COVID-19-associated GBS has a few clinical characteristics that increase suspicion of diagnosis in addition to the attributes of classic GBS. Furthermore, the response to plasmapheresis, intravenous immunoglobulin, elevated protein in CSF, and lack of COVID-19 virus detection in CSF may suggest an immune response rather than an inflammatory or infectious one [13].

## 4. Conclusion

Any patient having sensory symptoms or weakness during or after the course of illness of COVID-19 should be evaluated to rule out the possibility of GBS. Patients diagnosed with GBS should be treated with intravenous immunoglobulin or plasmapheresis along with standard supportive care and ICU care. Further studies are required for a better understanding of the relationship between SARS-COVID-19 infection and GBS.

## Figures and Tables

**Table 1:** Nerve conduction study findings in our patient.

Sensory						
Nerve/sites	Recording site	Amp (*µ*V)	Reference range (mean ± SD)	Velocity (m/s)	Reference range	

*Left sural*						
Calf	Lateral malleolus	11.7	19 ± 7	40.7	>40	

*Right sural*						
Calf	Lateral malleolus	21.3	19 ± 7	53	>40	

*Right radial (superficial)*						
Snuff box	Forearm	15.5	48 ± 15	48.6	>40	

*Motor NCS*						
Nerve/sites	Latency (ms)	Reference range	Amp. (mV)	Reference range (mean ± SD)	Velocity (m/s)	Reference range

*L tibial malleolus-AH*						
Ankle	6.70	<6	3.5	11.6 ± 4.3		
Popliteal fossa	19.14		2.8		29.7	>40

*Right median-APB*						
Wrist	8.0	<3.5	3.4	9.5 ± 2.7		
Elbow	16		3.4		26	>50

*F-waves*						
Nerve	Min. F latency (ms)	Reference range (mean ± SD)	Persist (%)			
Left tibial-AH	81.84	52.3 ± 4.3	100			
Right median-APB	67	29.1 ± 2.3	20			

## Data Availability

The authors declare that data supporting the findings of this case study are available within the article.
